# Balance diagnostics for comparing the distribution of baseline covariates between treatment groups in propensity-score matched samples

**DOI:** 10.1002/sim.3697

**Published:** 2009-09-15

**Authors:** Peter C Austin

**Affiliations:** 1 Institute for Clinical Evaluative SciencesG1 06, 2075 Bayview Avenue, Toronto, Ontario, Canada; 2 Dalla Lana School of Public Health, University of TorontoToronto, Ontario, Canada; 3 Department of Health Policy, Management and Evaluation, University of TorontoCanada

**Keywords:** balance, goodness-of-fit, observational study, propensity score, matching, propensity-score matching, standardized difference, bias

## Abstract

The propensity score is a subject's probability of treatment, conditional on observed baseline covariates. Conditional on the true propensity score, treated and untreated subjects have similar distributions of observed baseline covariates. Propensity-score matching is a popular method of using the propensity score in the medical literature. Using this approach, matched sets of treated and untreated subjects with similar values of the propensity score are formed. Inferences about treatment effect made using propensity-score matching are valid only if, in the matched sample, treated and untreated subjects have similar distributions of measured baseline covariates. In this paper we discuss the following methods for assessing whether the propensity score model has been correctly specified: comparing means and prevalences of baseline characteristics using standardized differences; ratios comparing the variance of continuous covariates between treated and untreated subjects; comparison of higher order moments and interactions; five-number summaries; and graphical methods such as quantile–quantile plots, side-by-side boxplots, and non-parametric density plots for comparing the distribution of baseline covariates between treatment groups. We describe methods to determine the sampling distribution of the standardized difference when the true standardized difference is equal to zero, thereby allowing one to determine the range of standardized differences that are plausible with the propensity score model having been correctly specified. We highlight the limitations of some previously used methods for assessing the adequacy of the specification of the propensity-score model. In particular, methods based on comparing the distribution of the estimated propensity score between treated and untreated subjects are uninformative. Copyright © 2009 John Wiley & Sons, Ltd.

## 1. INTRODUCTION

Researchers are increasingly using observational studies to estimate the effects of treatments and exposures on health outcomes. In randomized controlled trials (RCTS), randomization ensures that, on average, treated subjects will not differ systematically from untreated subjects in both measured and unmeasured baseline characteristics. Non-randomized studies of the effect of treatment on outcomes can be subject to treatment-selection bias in which treated subjects differ systematically from untreated subjects.

Propensity-score methods are being used with increasing frequency to estimate treatment effects using observational data. The propensity score is defined as the probability of treatment assignment conditional on measured baseline covariates [1, 2]. Rosenbaum and Rubin demonstrated a key property of the propensity score: conditional on the true propensity score, treatment status is independent of measured baseline covariates [1]. In other words, treated and untreated subjects with the same propensity score will have similar distributions of observed baseline covariates.

Four methods of using the propensity score have been described in the statistical literature: covariate adjustment using the propensity score, stratification on the propensity score, propensity-score matching, and inverse probability of treatment weighting using the propensity score [3]. In the medical literature, the first three are used almost exclusively [4–6]. Propensity-score matching, which is the focus of the current paper, is used frequently in the medical literature [7–9]. It entails forming matched sets of treated and untreated subjects with similar values of the propensity score. The most common implementation of propensity-score matching is 1:1 matching in which matched pairs of treated and untreated subjects are formed.

Rubin argues that an advantage to the use of propensity-score methods is that it allows observational studies to be designed similar to randomized experiments: the design of the study is separated from the analysis of the effect of exposure on the outcome [10]. Rubin states that ‘diagnostics for the successful design of observational studies proposed on estimated propensity scores … is a critically important activity in most observational studies’ [11]. In randomized or designed experiments, the propensity score is frequently known and fixed by the study design. However, in observational studies, the true propensity score is not known and must be estimated using the study data. The propensity score is a balancing score: conditional on the *true* propensity score, treated and untreated subjects have the same distribution of observed baseline covariates. Ho *et al.* have defined the propensity-score tautology as follows: ‘we know we have a consistent estimate of the propensity score when matching on the propensity score balances the raw covariates’ [12]. In other words, Ho *et al.* are suggesting that one has adequately specified the propensity-score model when, after matching on the estimated propensity score, the distribution of measured baseline covariates is similar between treated and untreated subjects. Therefore, the appropriateness of the specification of the propensity score is assessed by examining the degree to which matching on the estimated propensity score has resulted in a matched sample in which the distribution of measured baseline covariates is similar between treated and untreated subjects.

We use the term balance diagnostics to describe methods for assessing whether the distribution of baseline covariates is similar between different exposure groups. In the context of propensity-score matching, balance diagnostics enable applied researchers to assess whether the propensity-score model has been adequately specified. The test of adequate specification is whether matching on the propensity score has removed observed systematic differences between treated and untreated subjects. Descriptions of balance diagnostics in the context of propensity-score matching are scattered across the statistical, medical, and social sciences literature.

The objective of the current study is three-fold. First, to summarize in one paper previously described methods for assessing balance in measured baseline covariates between treated and untreated subjects in propensity-score matched samples. Second, to provide a framework for assessing the adequacy of the specification of the propensity score model. Third, to highlight limitations of some previously suggested methods for assessing the adequacy of the specification of the propensity-score model. The paper is structured as follows. In Section 2, we describe the data that were used for illustrating the different methods for assessing balance in baseline characteristics. In Section 3, we describe methods for comparing the mean of continuous variables or the prevalence of dichotomous baseline covariates between treated and untreated subjects in the propensity-score matched sample. In Section 4, we use Monte Carlo simulations to illustrate that obtaining balance in means alone can be insufficient. We then describe additional methods for comparing the distribution of baseline covariates between treated and untreated subjects in the matched sample. In Section 5, we highlight limitations of some previously proposed methods for assessing balance. Finally, in Section 6, we summarize our findings.

## 2. DATA SOURCES AND PROPENSITY-SCORE MATCHING METHODS

### 2.1. Data sources

We used data on 9104 patients who were discharged alive following hospitalization with a diagnosis of acute myocardial infarction (AMI or heart attack) from 102 hospitals in Ontario, Canada, between April 1, 1999 and March 31, 2001. These data are similar to those reported on elsewhere [13–15], and were collected as part of the Enhanced Feedback for Effective Cardiac Treatment (EFFECT) Study, an initiative focused on improving the quality of care for cardiovascular disease patients in Ontario [16]. Data on patient demographics, presenting signs and symptoms, classic cardiac risk factors, comorbid conditions and vascular history, vital signs on admission, and results of laboratory tests, were abstracted directly from patients’ medical records. The exposure of interest was whether the patient was prescribed a statin at hospital discharge. Overall, 3049 (33.5 per cent) of patients received a prescription for a statin at discharge, while 6055 (66.5 per cent) did not receive a prescription at discharge. Table I compares the means of continuous baseline covariates and prevalences of dichotomous baseline covariates between treated and untreated subjects in the original unmatched sample. The prevalence of dichotomous variables was compared between treated and untreated subjects using a Chi-squared test, while a standard two-sample *t*-test was used to compare continuous baseline covariates.

**Table I:** Comparison of baseline characteristics between treated and untreated subjects in the original unmatched sample

Variable	Statin: no (*N* = 6055)	Statin: yes (*N* = 3049)	*P* -value	Standardized differences
*Demographic characteristics*				
Age	68.1±13.8	63.4±12.4	<0.001	0.355
Female	2241 (37.0 Per cent)	887 (29.1 Per cent)	<0.001	0.167
*Presenting signs and symptoms*				
Acute CHF/pulmonary edema	316 (5.2 Per cent)	122 (4.0 Per cent)	0.010	0.057
Cardiogenic shock	46 (0.8 Per cent)	12 (0.4 Per cent)	0.038	0.046
*Cardiac risk factors*				
Diabetes	1561 (25.8 Per cent)	774 (25.4 Per cent)	0.684	0.009
Current smoker	2004 (33.1 Per cent)	1070 (35.1 Per cent)	0.057	0.042
Hyperlipidemia	1138 (18.8 Per cent)	1761 (57.8 Per cent)	<0.001	0.910
Hypertension	2681 (44.3 Per cent)	1453 (47.7 Per cent)	0.002	0.068
Family history of CAD	1762 (29.1 Per cent)	1177 (38.6 Per cent)	<0.001	0.204
*Comorbid conditions*				
Cerebrovascular disease/TIA	610 (10.1 Per cent)	237 (7.8 Per cent)	<0.001	0.079
Angina	1869 (30.9 Per cent)	1086 (35.6 Per cent)	<0.001	0.102
Cancer	191 (3.2 Per cent)	73 (2.4 Per cent)	0.041	0.045
Chronic CHF	275 (4.5 Per cent)	91 (3.0 Per cent)	<0.001	0.079
Renal disease	34 (0.6 Per cent)	13 (0.4 Per cent)	0.396	0.019
*Vital signs on admission*				
Systolic blood pressure	148.7±31.6	149.3±30.1	0.338	0.021
Diastolic blood pressure	83.6±18.6	84.5 ±18.0	0.033	0.047
Heart rate	84.6 ±24.3	81.7±23.0	<0.001	0.121
Respiratory rate	21.2±5.7	20.3±4.8	<0.001	0.167
*Laboratory tests*				
Glucose	9.4±5.1	9.2±5.3	0.092	0.037
White blood count	10.3±4.9	10.0±4.4	0.003	0.065
Hemoglobin	137.5±19.3	140.6±16.9	<0.001	0.167
Sodium	138.9±3.9	139.2±3.3	<0.001	0.079
Potassium	4.1±0.6	4.1 ±0.5	0.006	0.061
Creatinine	105.7±65.5	99.9±50.0	<0.001	0.096

*Notes:* Continuous variables are reported as mean ± standard deviation. Dichotomous variables are reported as *N* (Per cent).

### 2.2. Propensity-score matching

The propensity score was estimated using logistic regression to regress receipt of a statin prescription at discharge on the 24 baseline covariates described in Table I. The estimated propensity score was the predicted probability of statin exposure derived from the fitted logistic regression model. In the propensity-score model we assumed a linear relationship between continuous covariates and the log-odds of receiving a statin prescription. Furthermore, the propensity-score model did not include any interactions.

We created a matched sample by matching treated and untreated subjects on the logit of the propensity score using calipers of width equal to 0.2 of the standard deviation of the logit of the propensity score [3, 17, 18]. A greedy, nearest-neighbour matching algorithm was employed to form pairs of treated and untreated subjects.

## 3. COMPARING THE MEAN OR PREVALENCE OF A BASELINE COVARIATE BETWEEN TREATED AND UNTREATED SUBJECTS IN THE PROPENSITY-SCORE MATCHED SAMPLE

### 3.1. Comparing summary statistics between treated and untreated subjects

The CONSORT statement suggests that no RCT should be published without a summary of baseline characteristics in the different randomization arms [19]. In an explanation and elaboration of the CONSORT statement, it is suggested that baseline information is ‘efficiently presented in a table’ [20]. Similarly, any study that employs propensity-score matching should examine and report the means and/or medians of continuous variables and the distribution of categorical variables in treated and untreated subjects in the matched sample. These crude comparisons allow both the investigator and readers to conduct a basic assessment of the comparability of the two groups in the matched sample. Descriptive comparisons of baseline covariates in propensity-score matched samples are frequently reported in the medical literature [7–9].

Table II reports the means of continuous variables and prevalences of dichotomous variables in both treated and untreated subjects in the propensity-score matched sample. One observes that matching on the propensity score has diminished or eliminated many of the systematic differences in means or prevalences between treated and untreated subjects that were reported in Table I.

**Table II:** Comparison of baseline characteristics between treated and untreated subjects in the propensity-score matched sample

Variable	Statin: no (*N* = 2430)	Statin: yes (*N* = 2430)	Standardized difference
*Demographic characteristics*			
Age	63.4±13.2	63.5± 12.6	0.011
Female	717 (29.50 Per cent)	736 (30.3 Per cent)	0.017
*Presenting signs and symptoms*			
Acute CHF/pulmonary edema	94 (3.9 Per cent)	93 (3.8 Per cent)	0.002
Cardiogenic shock	15 (0.6 Per cent)	12 (0.5 Per cent)	0.017
*Cardiac risk factors*			
Diabetes	609 (25.1 Per cent)	606 (24.9 Per cent)	0.003
Current smoker	883 (36.3 Per cent)	872 (35.9 Per cent)	0.009
Hyperlipidemia	1135 (46.7 Per cent)	1,142 (47.0 Per cent)	0.006
Hypertension	1109 (45.6 Per cent)	1,118 (46.0 Per cent)	0.007
Family history of CAD	898 (37.0 Per cent)	912 (37.5 Per cent)	0.012
*Comorbid conditions*			
Cerebrovascular disease/TIA	185 (7.6 Per cent)	187 (7.7 Per cent)	0.003
Angina	830 (34.2 Per cent)	810 (33.3 Per cent)	0.017
Cancer	66 (2.7 Per cent)	61 (2.5 Per cent)	0.013
Chronic CHF	78 (3.2 Per cent)	70 (2.9 Per cent)	0.019
Renal disease	11 (0.5 Per cent)	10 (0.4 Per cent)	0.006
*Vital signs on admission*			
Systolic blood pressure	149.1 ±30.2	149.3±30.2	0.007
Diastolic blood pressure	84.3±18.1	84.6±18.4	0.015
Heart rate	81.3±22.5	81.6±22.8	0.014
Respiratory rate	20.3 ±4.8	20.3 ±4.7	0.012
*Laboratory tests*			
Glucose	9.2±5.1	9.3±5.5	0.017
White blood count	10.2±4.1	10.1 ±4.7	0.006
Hemoglobin	140.7±18.2	140.5 ±17.0	0.007
Sodium	139.2±3.7	139.1±3.3	0.025
Potassium	4.1 ±0.5	4.1±0.5	0.030
Creatinine	100.5 ±59.0	100.5 ±52.4	0.000

*Notes:* Continuous variables are reported as mean ± standard deviation. Dichotomous variables are reported as *N* (Per cent).

### 3.2. Standardized differences for comparing means and prevalences between groups

For continuous variables, the standardized difference is defined as


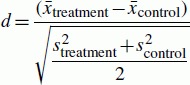
(1)

where 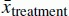 and 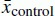 denote the sample mean of the covariate in treated and untreated subjects, respectively, while 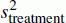 and 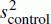 denote the sample variance of the covariate in treated and untreated subjects, respectively. For dichotomous variables, the standardized difference is defined as


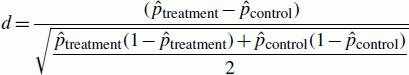
(2)

where 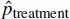 and 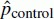 denote the prevalence or mean of the dichotomous variable in treated and untreated subjects, respectively.

The standardized difference compares the difference in means in units of the pooled standard deviation [21]. Unlike *t*-tests and other statistical tests of hypothesis, the standardized difference is not influenced by sample size. Thus, the use of the standard difference can be used to compare balance in measured variables between treated and untreated subjects in the matched sample with that in the unmatched sample. Furthermore, it allows for the comparison of the relative balance of variables measured in different units (e.g. age in years with systolic blood pressure in mmHg). In an earlier article on matching on the propensity score, Rosenbaum and Rubin use the standardized difference to assess the comparability of treated and untreated subjects in matched samples [22]. Since then, several authors have used this approach in the clinical literature [7, 9, 23].

**Figure 1:**
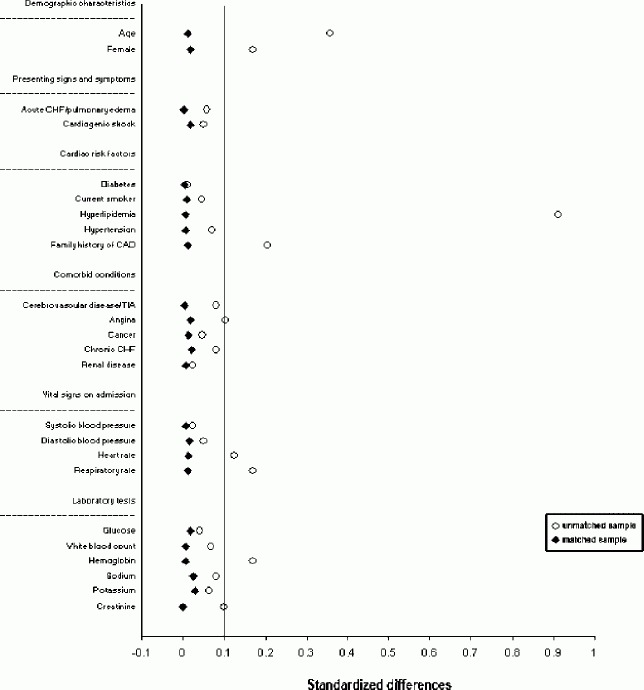
Absolute standardized differences for baseline covariates comparing treated to untreated subjects in the original and the matched sample.

Absolute standardized differences comparing baseline covariates between treated and untreated subjects in the unmatched and matched sample are reported in Tables I and II, respectively. In the unmatched sample the largest absolute standardized difference was for a history of hyperlipidemia (0.910). In contrast, in the matched sample the largest absolute standardized difference was for potassium (0.030). One observes that matching on the propensity score resulted in a matched sample in which the means and prevalences of baseline covariates are very similar between treated and untreated subjects. Several papers in the cardiology literature have reported standardized differences in both the original and matched sample using graphical displays that first appear to have been developed by Love and colleagues [24, 25]. Figure 1 reports absolute standardized differences in a similar graphical manner.

The standardized difference was proposed in the psychological literature, where it has been referred to as Cohen's Effect Size Index [26]. Cohen suggested that Effect Size Indices of 0.2, 0.5, and 0.8 can be used to represent small, medium, and large effect sizes, respectively [26]. When the two populations being considered are normally distributed with equal variance and are of the same size, Cohen derived relationships between the Effect Size Index and the percentage of non-overlap of the two distributions (*U*_1_), the percentage in the second population that exceeds the same percentage in the first population (*U*_2_), and the percentage of the first population which the upper half of the second population exceeds (*U*_3_) [26]. Let *d* denote the Effect Size Index and be taken to represent a deviate from the standard normal distribution. Furthermore, let *F(d)* denote the cumulative normal distribution function for the deviate *d*. Then Cohen derived the following relationships between the Effect Size Index and the three different measures of overlap between the two population distributions:


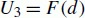
(3)


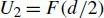
(4)


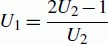
(5)

For instance, when the Effect Size Index (*d*) is equal to 0.1, then *U*_1_, *U*_2_, and *U*_3_ are equal to 7.7 per cent, 52.0 per cent, and 54.0 per cent, respectively. Thus, when the standardized difference is equal to 0.10, the percentage of non-overlap between the distributions of the continuous covariate in the two groups is 7.7 per cent.

Cohen also derived relationships between the Effect Size Index (*d*) and the Pearson correlation coefficient (*r*) between the covariate and a dichotomous variable denoting group membership:


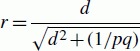
(6)

where *p* denotes the proportion of the combined population that is in the first population and *q* is equal to 1 − *p*. Given *r*, one can calculate *r*^2^, the proportion of the variance in the covariate that is explained by group membership. Given two equally sized populations and an Effect Size Index of 0.1, then the Pearson correlation coefficient is 0.05. This implies that group membership explains 0.25 per cent of the variance of the covariate. In our matched sample, the largest standardized difference was 0.03 (potassium). The Pearson correlation coefficient would be 0.015, and group membership explains 0.0225 per cent of the variance in potassium.

Standardized differences are increasingly being used to compare balance in baseline covariates between treated and untreated subjects in the propensity-score matched sample. A limitation to their use is lack of consensus as to what value of a standardized difference denotes important residual imbalance between treated and untreated subjects in the matched sample. While there is no clear consensus on this issue, some researchers have proposed that a standardized difference of 0.1 (10 per cent) denotes meaningful imbalance in the baseline covariate [23]. It is likely that the threshold for acceptable imbalance depends, to some degree, on the prognostic importance of the covariate in question. Ho *et al.* suggest that better balance is more important for prognostically important covariates than for weak predictors of the outcome [12].

### 3.3. The empirical sampling distribution of the standardized difference

In this subsection, we propose methods to estimate the empirical sampling distribution of the standardized difference of the mean under the assumption that the mean (or prevalence) of a covariate is equal between two groups. This allows one to determine a plausible range of observed standardized differences when the propensity score model has been correctly specified.

We first consider the case of a continuous covariate and two independent samples. Assume that the true standardized difference is equal to δ Then the large sample distribution of the standardized differences converges to a normal distribution with mean δ and variance (*n_T_* + *n_C_*)/*n_T_n_C_* + δ/2(*n_T_*+*n_C_*) [27], where *n_C_* and *n_T_* denote the number of untreated and treated subjects, respectively. Under the assumption that the true standardized difference is zero (δ = 0), then the variance of the standardized difference is equal to (*n_T_* + *n_C_*)/*n_T_n_C_*. If we make the further assumption that the two groups have equal number of subjects (*n_T_* = *n_C_* = *n*), then the variance of the standardized difference is equal to 2/*n*. Therefore, the standard deviation of the standardized difference is 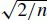. Thus, in 95 per cent of samples drawn from a population in which the true standardized difference is zero, the estimated standardized difference will lie within ±1.962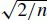.

There are important implications of the above results. First, in RCTs, balance of means of covariates is a large sample property. While randomization will, on average, result in measured and unmeasured covariates being balanced between treatment arms of the study, this need not be true in any particular study. Second, in small randomized trials, one is more likely to observe systematic differences in baseline covariates between treated and untreated subjects than in large randomized trials.

The sampling distribution of the standardized difference for a binary covariate and two independent samples has not, to our knowledge, been derived. Furthermore, the above result assumes that the two samples are independent of one another. However, propensity-score matching induces a lack of independence. Matched subjects have similar propensity scores. Therefore, they have measured baseline covariates that come from the same multivariate distribution [1]. Thus, baseline covariates within a propensity-score matched sample are likely to be correlated (it is for this reason variance estimation of the estimated treatment effect should account for the matched-pairs nature of the sample [28]).

We propose a resampling-based approach to deriving the empirical sampling distribution of either a continuous or a binary covariate in propensity-score matched samples. Under the null hypothesis of a standardized difference equal to zero (equivalent to equality of means or proportions), the within-pair values of the covariate are exchangeable. Therefore, one can randomly permute the within-pair values of the covariate and then compute the standardized difference. This process can be repeated a large number of times (we used 1000 repetitions). Then, the empirical sampling distribution of the standardized difference can be determined under the assumption that the true standardized difference is zero.

In our case study, we considered 11 continuous covariates. The standard deviation of the empirical sampling distribution for the standardized difference of age was 0.017. Thus, the 2.5th percentile of the sampling distribution of the standardized difference of age under the null hypothesis would be approximately −0.033, while the 97.5th percentile would be approximately 0.033. For the 10 remaining continuous covariates, the standard deviation of the empirical sampling distribution ranged from 0.024 to 0.029. Thus, the 2.5th percentile of the sampling distribution under the null hypothesis would range from approximately −0.057 to −0.047, while the 97.5th percentiles would range from 0.047 to 0.057. In examining Table II, one observes that the estimated standardized differences for the 11 continuous covariates are at most 0.030 (in absolute value).

We considered 13 binary covariates in our case study. For history of hyperlipidemia, the standard deviation of the empirical sampling distribution of the null hypothesis was approximately 0.003. Thus, the 2.5th and 97.5th percentiles of the sampling distribution would be approximately −0.006 and 0.006. For the remaining 12 binary covariates, the standard deviation of the empirical sampling distributions of the standardized differences ranged from 0.026 to 0.029. Thus, the 2.5th percentile of the sampling distribution under the null hypothesis would range from approximately −0.057 to −0.051, while the 97.5th percentiles would range from 0.051 to 0.057. As with the continuous variables, for all of the binary covariates, the estimated standardized differences in Table II lie within the 2.5th and 97.5th percentiles. Therefore, there is no evidence that the propensity score model has been incorrectly specified.

**Figure 2:**
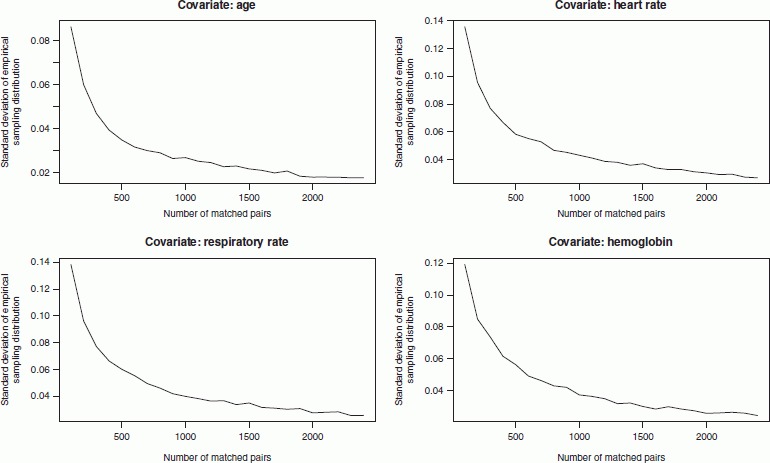
Relationship between sample size and the standard deviation of empirical sampling distribution of standardized difference.

We conducted an additional set of analyses to illustrate the influence of sample size on the variability of the estimated standardized difference. We selected four continuous covariates: age, heart rate, respiratory rate, and hemoglobin. We then selected a random sample of matched pairs. We allowed the number of matched pairs to increase from 100 to 2400 in increments of 100. Within each random sample of matched pairs, we used the method described above to estimate the standard deviation of the empirical sampling distribution of the standardized difference of the mean. The relationship between the standard deviation of the empirical sampling distribution and the number of matched pairs is described in Figure 2. This figure clearly illustrates the inverse relationship between sample size and standard deviation of the empirical sampling distribution of the standardized difference of the mean. When the number of matched pairs is small, there are a wide range of standardized differences that are consistent with the propensity-score model having been correctly specified. For instance, if there were only 100 matched pairs, then the 2.5th and 97.5th percentiles for the sampling distribution of the standardized difference of the mean for hemoglobin would be approximately −0.234 and 0.234, respectively. If the number of matched pairs increased to 200, then these percentiles would change to −0.166 and 0.166, respectively. Thus, in small matched samples, moderate standardized differences could still be consistent with the propensity-score model having been correctly specified. This figure illustrates the concept that in observational studies, as in RCTs, balance is a large-sample property; moderate imbalance can be expected in small samples, even if the propensity score model has been correctly specified.

## 4. COMPARING VARIANCES, INTERACTIONS, AND DISTRIBUTIONS BETWEEN TREATMENT GROUPS IN PROPENSITY-SCORE MATCHED SAMPLES

In the previous section, we discussed methods for comparing the mean and prevalences of measured baseline covariates between treated and untreated subjects in propensity-score matched samples. However, conditioning on the propensity score does not just balance means between treated and untreated subjects. More generally, subjects matched on the propensity score will have the same distribution of measured baseline covariates [1]. Given a baseline covariate *X*, Rosenbaum and Rubin suggest that if the outcome has a non-linear regression on *X* in each of the treated and untreated groups, then there can exist bias due to *X* in the matched sample even if the mean of *X* is the same in treated and untreated subjects in the matched sample [22]. In Section 4.1, we present a limited set of Monte Carlo simulations to illustrate that one should examine balance in higher order moments and interactions, and not only in means. Then, in Section 4.2, we propose additional balance diagnostics for comparing the distribution of measured baseline covariates between treated and untreated subjects in the matched sample. We describe both quantitative and qualitative methods for assessing balance in observed baseline covariates between treated and untreated subjects in the matched sample. The use of each method is illustrated using the data described in Section 2.

### 4.1. Monte Carlo simulations

We conducted three sets of Monte Carlo simulations to demonstrate that when the true propensity score involves either quadratic terms or interactions, then matching on a mis-specified propensity score can result in balancing means between treated and untreated subjects. However, bias due to confounding remains compared with when the correctly specified propensity-score model was used.

#### 4.1.1. Methods

In the first of simulations (Scenario A), a random variable *X*_1_ was generated from a standard normal distribution for each of 1000 subjects (*X*_1_∼N(0,1)). A new variable, *X*_2_, was defined: 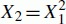. The logit of the probability of treatment assignment was determined from the following model: 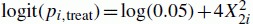. For each subject, treatment status was randomly generated from a Bernoulli distribution with subject-specific parameter *p*_*i*, treat_. Then, for each subject, a continuous outcome was generated from the following model: 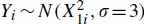. Thus, the outcome is independent of treatment status. Furthermore, variation in *X*_1_ explains 10 per cent of the variation in the outcome *Y.* Two different propensity-score models were fit. The first contained only *X*_1_ as a predictor variable, while the second contained both *X*_1_ and 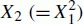 as predictors (thus the first was mis-specified, while the second was correctly specified). Subjects were matched on the logit of the estimated propensity score using calipers of width equal to 0.2 of the standard deviation of the logit of the propensity score. The absolute standardized difference comparing the mean of *X*_1_ and *X*_2_ between treated and untreated subjects was computed. We also determined the ratio of the variance of *X*_1_ and *X*_2_ in treated subjects to that in untreated subjects. The difference in the mean of *Y* between treated and untreated subjects was determined in the matched sample. This process was repeated 1000 times and the results were averaged over the 1000 simulations.

In the second set of simulations (Scenario B), two independent standard normal random variables were generated for each of 1000 subjects (*X*_1_∼N(0,1) and *X*_2_∼N(0,1)). We defined *X*_3_ to be the product of these two random variables. The logit of the probability of treatment assignment was defined using the following model: logit(*p*_*i*,treat_) = log(1/3) + *X*_1,*i*_ + *X*_2,*i*_ + *X*_3,*i*_. For each subject, a treatment status was randomly generated from a Bernoulli distribution with subject-specific parameter *p*_*i*,treat_. Then, for each subject, a continuous outcome was generated as follows: *Y_i_*∼N(*X*_1,*i*_ + *X*_2,*i*_ + *X*_3,*i*_, σ = 4.48) (using a standard deviation of 4.48 implies that variation in the covariates explains 13 per cent of the variation in *Y*). Two propensity-score models were fit to the randomly generated sample. The first propensity-score model contained only *X*_1_ and *X*_2_ as predictors, while the second propensity-score model was the correctly specified propensity-score model. Propensity-score matching was done as described in Scenario A. Computations similar to those in Scenario A were conducted in the matched sample. This process was repeated 1000 times and the results were averaged across the 1000 simulated data sets.

The third set of simulations (Scenario C) was identical to Scenario B, except that *X*_2_ was generated from a Bernoulli distribution with parameter 0.5. Thus, *X*_3_ was an interaction between a continuous covariate and a binary indicator variable.

#### 4.1.2. Results

The results of the Monte Carlo simulations are reported in Table III. In Scenario A, the standardized difference comparing the mean of *X*_1_ between treated and untreated subjects in the matched sample was low, regardless of which specification of the propensity-score model was used. However, the ratio of the variance of *X*_1_ in the treated subjects to the variance of *X*_1_ in untreated subjects was further from one when the propensity-score model was mis-specified, compared with when the propensity-score model was correctly specified. Similarly, the standardized difference of 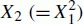 was moderately larger when the propensity-score model was mis-specified compared with when it was properly specified.

**Table III:** Results of simulations examining balance in matched sample when propensity score model is mis-specified compared to when it is properly specified

						
	Scenario A		Scenario B		Scenario C	
			
Statistic	Propensity-score model incorrectly specified	Propensity-score mode correctly specified	Propensity-score model incorrectly specified	Propensity-score model correctly specified	Propensity-score model incorrectly specified	Propensity-score model correctly specified
*Balance diagnostics*						
*X*_1_-standardized difference	0.01	0.05	0.04	0.04	0.02	0.03
*X*_1_-variance ratio	1.14	1.04	0.65	1.02	0.77	1.02
*X*_2_-standardized difference	0.22	0.07	0.04	0.04	0.04	0.04
*X*_2_-variance ratio	1.43	1.12	0.65	1.02	NA	NA
*X*_3_-standardized difference	NA	NA	0.45	0.05	0.23	0.03
*X*_3_-variance ratio	NA	NA	0.46	1.08	0.89	1.05
*Estimated treatment effect (difference in means between treated and untreated subjects in matched sample)*						
Difference in *Y*	0.08	0.04	0.35	0.02	0.11	0.02

*Notes:* Variance ratios are the mean ratio of the variance of a variable in treated subjects to the variance of the variable in untreated subjects.

In Scenario B, the mean absolute standardized difference for both *X*_1_ and *X*_2_ were small, regardless of the specification of the propensity-score model. However, then mean absolute standardized difference for *X*_3_ was substantially larger when the propensity-score model was mis-specified compared with when it was correctly specified. Furthermore, the mean ratio of the variance of *X*_1_ in treated subjects to the variance in untreated subjects was further from unity when the propensity-score model was mis-specified, compared with when it was properly specified. Similar findings were observed for the variance of *X*_2_ and *X*_3_.

In Scenario C, the mean absolute standardized difference for both *X*_1_ and *X*_2_ was small, regardless of whether the propensity-score model was correctly specified. However, the absolute standardized difference for *X*_3_ was moderately larger when the propensity-score model was mis-specified, compared with when it was correctly specified. Furthermore, the mean ratio of the variance of *X*_1_ in treated subjects to the variance in untreated subjects was further from unity when the propensity-score model was mis-specified, compared with when it was properly specified. Similar findings were observed for the variance of *X*_2_ (we did not examine the variance of *X*_2_ since it is a binary variable).

In all three scenarios, one observes that the bias in estimating the treatment effect increases when the propensity-score model was incorrectly specified.

#### 4.1.3. Summary

Our set of three Monte Carlo simulations demonstrated that observing balance on the means of covariates does not imply that the propensity-score model has been correctly specified. Greater bias was observed when matching on the mis-specified propensity score compared with when matching on the correctly specified propensity score despite the fact that balance in the means of covariates was observed. Thus, observing balance on means of main effects alone does not ensure that bias has been eliminated from estimating the treatment effect. However, within each of the three scenarios, the ratio of the variance of a baseline covariate in treated subjects compared with that in untreated subjects was moderately different from one when the propensity- score model was mis-specified. In contrast, this ratio was close to one when the propensity-score model was correctly specified. Similarly, comparing the mean of interactions allowed one to determine whether the propensity-score model had been incorrectly specified. This finding suggests that balance diagnostics needs to incorporate methods for comparing the distribution of measured baseline covariates between treated and untreated subjects in the matched sample.

### 4.2. Methods for comparing distributions of baseline covariates

In this section we describe methods to complement the comparison of means between treatment groups. When used as an adjuvant to the comparison of means, these methods allow one to compare whether the distribution of a baseline covariate is similar between treatment groups.

#### 4.2.1. Variance ratios

Imai *et al.* suggest that rather than limit comparisons to means, one also compares higher order moments of baseline covariates between treated and untreated subjects [29]. The variance is the second central moment. Similarly, Ho *et al.* suggest that standard deviations of continuous variables be compared between groups in the matched sample [12]. By comparing variances, in addition to means, between the two groups, one can obtain a broader characterization of the similarity of the distribution of a continuous covariate between two groups. In the Monte Carlo simulations described in Section 4.1, we illustrated that comparing ratios of variances can allow one to determine whether the propensity-score model has been incorrectly specified.

The variances of continuous baseline covariates in treated and untreated subjects and the ratio of these variances in both the unmatched and matched sample are reported in Table IV. For 9 of the 11 continuous covariates, matching on the propensity score resulted in variance ratios that were closer to unity compared with the ratios in the unmatched sample. For instance, in the unmatched sample, the variance of age was 20 per cent less in treated subjects than it was in untreated subjects. However, in the matched sample, the variance of age was only 10 per cent less in treated subjects than it was in untreated subjects. The two exceptions were white blood count and glucose. For these two covariates, the discrepancies in the variances between treated and untreated subjects were greater in the matched sample than in the unmatched sample.

Under the null hypothesis of equality of variances of a continuous variable between two independent samples, the distribution of the estimated variances follows an *F*-distribution [30]. While the distribution of the ratio of variances in dependent samples is not known, the percentiles of the *F*-distribution can be used as a rough guide as to what variances ratios are consistent with equality of variances in the two groups. Our matched sample consisted of 2430 matched pairs. The 2.5th and 97.5th percentiles of the *F*-distribution with 2429 and 2429 degrees of freedom are 0.92 and 1.08, respectively. In examining Table IV, one notes that 6 of the 11 continuous variables have variances ratios that exceed these thresholds. The most extreme variance ratio was for sodium (0.77).

**Table IV:** Variances of continuous covariates in treated and untreated subjects in unmatched and matched samples

	Unmatched sample			Matched sample		
		
Variable	Variance (untreated subjects)	Variance (treated subjects)	Ratio: treated to untreated variances	Variance (untreated subjects)	Variance (treated subjects)	Ratio: treated to untreated variances
Age	191.71	153.54	0.80	174.84	158.08	0.90
Heart rate	590.84	527.08	0.89	508.03	521.28	1.03
Systolic BP	996.68	904.15	0.91	914.95	909.29	0.99
Diastolic BP	346.7	323.95	0.93	327.17	337.04	1.03
Respiratory rate	32.9	22.81	0.69	23.32	22.09	0.95
WBC	23.75	19.51	0.82	17.2	21.64	1.26
Hemoglobin	374.32	286.34	0.76	331.62	288.36	0.87
Sodium	15.37	10.82	0.70	13.96	10.75	0.77
Glucose	26.1	28.05	1.08	26.04	30.77	1.18
Potassium	0.32	0.26	0.82	0.28	0.26	0.94
Creatinine	4283.81	2503.02	0.58	3477.95	2745.08	0.79

We then modified the propensity-score model by using restricted cubic splines to model the relationship of the six continuous variables (age, white blood count, haemoglobin, sodium, glucose, and creatinine) [31]. Subjects were then matched using the modified propensity score. In the resultant matched sample, the variance ratios for 10 of the 11 continuous variables lay between 0.93 and 1.03. The one exception was sodium, whose variance ratio was 0.91.

#### 4.2.2. Five-number summaries

The standardized difference described in Section 3 allows for the comparison of means and prevalences of baseline covariates between treated and untreated subjects in a propensity-score matched sample. However, the mean is one of many possible ways to summarize a distribution. Hoaglin *et al.* suggest that the five-number summary (minimum, first quartile, median, third quartile, maximum) provides about the right amount of detail for summarizing a distribution [32]. In strata matched on the true propensity score, treated and untreated subjects have similar distributions of baseline covariates. The use of five-number summaries allows a broader comparison of the distribution of a continuous covariate between treated and untreated subjects in the propensity-score matched sample. We are not aware of five-number summaries having been used in the context of propensity-score matching.

Table V reports the five-number summaries for each continuous baseline covariate in the matched sample in treated and untreated subjects separately. In comparing the five-number summaries between treated and untreated subjects in the unmatched sample, one observes that treated subjects tended to be younger than untreated subjects. However, the distribution of both systolic and diastolic blood pressure was comparable between the two groups in the unmatched sample. In the matched sample, the distribution of age was similar between treated and untreated subjects, although there may be some indication that the spread of the distribution was modestly greater in untreated patients compared with in treated patients.

**Table V:** Five-number summaries of continuous variables comparing treated and untreated subjects in both unmatched and matched samples

	Untreated subjects					Treated subjects				
		
Variable	Minimum	25th percentile	Median	75th percentile	Maximum	Minimum	25th percentile	Median 75th percentile	Maximum	
*Unmatched sample*										
Age	21	58	70	79	100	22	54	64	73	92
Heart rate	0	68	81	98	260	4	66	78	93	260
Systolic BP	0	128	148	170	266	0	130	149	169	270
Diastolic BP	0	70	83	96	199	0	72	84	96	182
Respiratory rate	5	18	20	23	60	5	18	20	20	58
WBC	0.3	7.6	9.5	12.1	98	0.76	7.6	9.4	11.5	90.1
Hemoglobin	39	127	139	151	199	58	131	142	152	199
Sodium	85	137	139	141	191	114	137	140	141	149
Glucose	1.2	6.4	7.8	10.7	89	1.4	6.3	7.6	10.2	92
Potassium	2	3.7	4.1	4.4	9.2	2.3	3.7	4	4.3	7
Creatinine	30	78	92	112	1151	20	77	91	108	926
*Matched sample*										
Age	21	53	64	74	96	22	54	64	74	92
Heart rate	0	66	78	94	230	12	66	78	93	210
Systolic BP	56	130	148	170	251	0	130	148	169	270
Diastolic BP	8	72	84	96	189	0	72	84	97	182
Respiratory rate	5	18	20	22	56	5	18	20	20	58
WBC	0.3	7.6	9.5	11.8	69.5	0.76	7.7	9.4	11.6	90.1
Hemoglobin	39	131	143	153	195	58	131	142	152	199
Sodium	85	138	140	141	191	114	137	140	141	149
Glucose	1.2	6.3	7.7	10.4	89	1.4	6.3	7.6	10.2	92
Potassium	2.4	3.7	4	4.3	7.3	2.3	3.7	4	4.3	7
Creatinine	30	77	90	106	1151	20	77	91	107	926

A limitation to the use of five-number summaries is that one does not know how much variation is reasonable if indeed the propensity-score model has been correctly specified. While the *F*-distribution can be used to describe the sampling variability of a ratio of variances, and the distribution of the standardized difference can be derived, comparable methods for five-number summaries are not available currently. The use of five-number may be best used as a rough, qualitative assessment of whether there has been gross mis-specification of the propensity-score model.

#### 4.2.3. Graphical summaries of the univariate distribution of continuous variables in treated and untreated subjects

We now describe graphical methods to qualitatively compare the univariate distribution of continuous baseline covariates between treated and untreated subjects in the weighted sample. Side-by-side boxplots [32], empirical cumulative distribution functions [33], quantile–quantile plots [32], and non-parametric estimates of density functions can be used to compare the univariate distribution of continuous baseline covariates between treated and untreated subjects in the matched sample. While standardized differences compare the difference in means between treated and untreated subjects, these graphical displays permit a broader comparison of a distribution of a continuous variable between two groups. Both Imai *et al.* and Ho *et al.* have proposed that quantile–quantile plots be used to compare the distribution of baseline covariates between treatment groups in a matched sample [12, 29].

**Figure 3:**
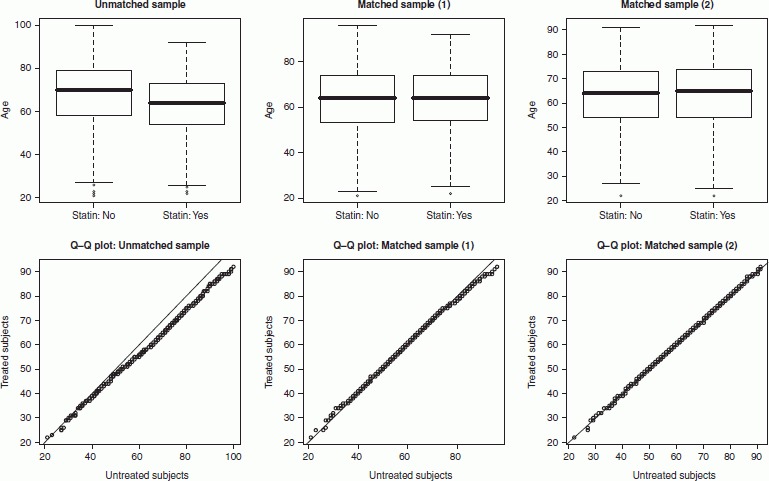
Side-by-side boxplots and *Q–Q* plots for age.

**Figure 4:**
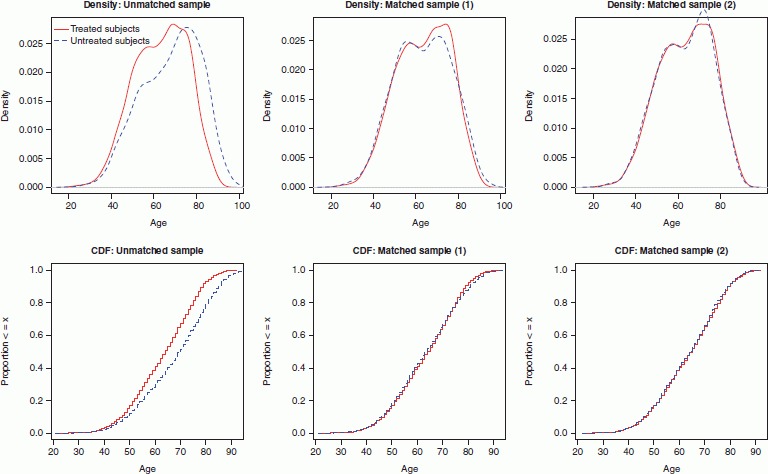
Density plots and cumulative distribution functions for age.

Figure 3 display side-by-side boxplots and quantile–quantile plots for age in both the unmatched and matched samples. We considered two different propensity-score models. The first was the originally specified propensity-score model. The second was the modification of initial model that was described in Section 4.2.1, in which the original propensity-score model was modified by using restricted cubic splines to model the relationship of the six continuous variables (age, white blood count, hemoglobin, sodium, glucose, and creatinine). Figure 4 displays non-parametric density plots and empirical cumulative distribution functions comparing the distribution of age between treated and untreated subjects in both the unmatched and matched samples. In examining Figure 3, one observes that matching on the initial propensity score has diminished differences in the distribution of age between treated and untreated patients. However, minor residual differences in the upper tail of the distribution persist in the matched sample. The side-by-side boxplots indicate that, in the matched sample, the distribution of age exhibits modestly greater variability in the untreated subjects than it does in the treated subjects. These observations are confirmed in examining the non-parametric density plots and empirical cumulative distribution functions displayed in Figure 4. Matching on the propensity score diminished differences in the distribution of age between treated and untreated subjects. However, slight differences in the distribution of age between the two groups persisted after matching. These residual differences in age between treated and untreated subjects were less apparent when means were compared (either directly or using standardized differences). The graphical comparisons indicate that the distribution of age is essentially identical between treated and untreated subjects in the sample obtained by matching on the modified propensity score. Thus, the graphical comparisons indicate that the modified propensity-score model is preferable to the original propensity-score model.

A limitation to the use of graphical measures for comparing the distribution of continuous baseline covariates between treated and untreated subjects is that it is difficult to assess the range of empirical comparisons that are consistent with the propensity-score model having been correctly specified. In a particular setting, one could use a resampling-based approach to determine the empirical sampling distribution of a given method for comparing distributions. However, one would need a simple way to summarize the variability in the graphical method.

#### 4.2.4. Interactions

In strata matched on the true propensity score, the multivariate distribution of observed baseline covariates will be similar between treated and untreated subjects (at least in large samples). Therefore, in large samples, one would expect that the covariance (or correlation) between pairs of variables to be similar between treated and untreated subjects. Ho *et al.* have suggested comparing interactions of pairs of variables between treated and untreated subjects in the propensity-score matched sample [12]. Comparing interactions allows one to determine if covariances and not just expectations or means are similar between two groups. In two of Monte Carlo simulations described in Section 3.1 (Scenarios B and C), we observed that the standardized differences for the interaction terms were large when the propensity-score model had been incorrectly specified; however, these same standardized differences were small when the propensity-score model was correctly specified.

In our sample there were 11 continuous baseline covariates, resulting in the consideration of 55 two-way interactions between continuous variables. For each of these interactions we computed the standardized difference of the interaction between treated and untreated subjects in the propensity-score matched sample. The 55 absolute standardized differences ranged from a low of 0.0003 to a high of 0.0274, with a median of 0.0098. The first and third quartiles were 0.0036 and 0.0175, respectively. Thus, there was at most negligible imbalance in interactions between pairs of continuous baseline variables between treated and untreated subjects in the matched sample.

In our sample there were 13 dichotomous baseline covariates. One can consider the 78 possible two-way interactions between these dichotomous covariates. Three of the standardized differences were undefined, resulting in 75 estimated standardized differences. The absolute value of these 75 standardized differences ranged from a low of 0 to a high of 0.048.

One can also consider the standardized differences for all two-way interactions between continuous and dichotomous variables. There were 143 possible interactions between the 11 continuous variables and the 13 dichotomous variables. The absolute value of the standardized differences ranged from a low of 0.000 to a high of 0.036, with a median of 0.003. Thus, there is no evidence of imbalance exceeding that which would be expected if the propensity-score model had been correctly specified.

## 5. LIMITATION OF PRIOR METHODS

### 5.1. Comparing the distributions of the estimated propensity score between treated and untreated subjects

Some authors have proposed that the distribution of the estimated propensity score be compared between treated and untreated subjects in the propensity-score matched sample [12, 29]. Side-by-side boxplots, empirical cumulative distribution functions, and non-parametric density estimates can be used to compare the distribution of the propensity score between treated and untreated subjects. In this subsection, we will demonstrate that these analyses may be uninformative.

**Figure 5:**
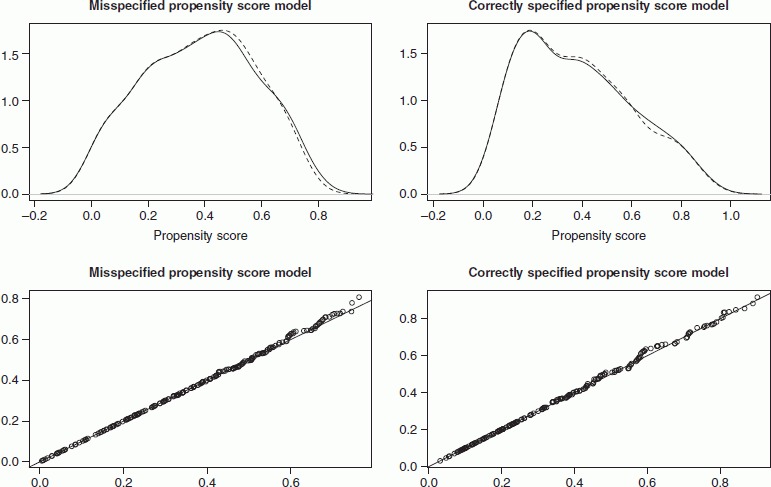
Distribution of estimated propensity score in treated and untreated subjects in different matched samples.

We examined one of the simulated data sets generated in Scenario B (in the true propensity-score model there exists an interaction between two continuous covariates) described in Section 4.1. The top two panels of Figure 5 describes non-parametric estimates of the distribution of the propensity score in treated and untreated subjects in the two propensity-score matched samples (mis-specified and correctly specified propensity-score models). Under both propensity score models, the distribution of the estimated propensity score was similar between treated and untreated subjects in the matched sample. The lower two panels of Figure 5 describe quantile–quantile plots comparing the distribution of the estimated propensity score in the two matched samples. Regardless of whether the propensity-score model was correctly specified, the quantile–quantile plots demonstrate the distribution of the estimated propensity score was essentially identical between treated and untreated subjects in the matched sample.

The above example illustrates that the similarity of the distribution of the estimated propensity score between treated and untreated subjects does not imply that measured baseline covariates have been balanced between treated and untreated subjects, nor does it imply that the propensity-score model has been correctly specified. However, these figures can be useful in describing the overlap in the distribution of the propensity score, to determine the degree of overlap between treated and untreated subjects. This can help inform the decision about the relative support prior to matching.

### 5.2. Significance testing

Significance testing has been frequently used to compare the distribution of measured baseline covariates between treated and untreated subjects in the propensity-score matched sample [7–9]. Imai *et al.* have argued that the use of significance testing to assess baseline balance in the matched sample is inappropriate for two reasons [29]. First, the sample size of the matched sample is less than that of the original unmatched sample. Thus, there is reduced statistical power to detect imbalance in the matched sample compared with in the original unmatched sample; apparent improvement in balance may be due to the reduced sample size. Second, Imai *et al.* suggest that balance is a property of a particular sample and makes no reference to a super-population. Imai *et* al.'s proscription against the use of significance testing has been echoed elsewhere [7, 8]. All of the methods described in Sections 3 and 4 are not influenced by sample size and are not based upon statistical hypothesis testing.

### 5.3. The c-statistic or area under the receiver operating characteristic (ROC) curve of the propensity-score model

Several applied studies report the ROC curve area (equivalent to the c-statistic) of the propensity-score model [6]. The ROC curve area measures the discrimination of the propensity-score model. However, two prior studies have found that the area under the ROC curve gives no indication of whether an important confounding variable had been omitted from the propensity-score model [17, 34]. In Scenario B of the Monte Carlo simulations considered in Section 4.1 (an interaction existed between two continuous predictors in the propensity-score model), the mean area under the ROC curve across the 1000 simulated data sets was 0.81 when the propensity-score model was mis-specified (by omitting the interaction), whereas it was 0.83 when the propensity-score model was correctly specified. Similarly, in Scenario C (an interaction existed between a continuous predictor and a dichotomous predictor in the propensity-score model), the mean area under the ROC curve was 0.820 when the propensity-score model was mis-specified, whereas it was 0.826 when the propensity-score model was correctly specified. These results suggest that one cannot rely on the area under the ROC curve to indicate whether the propensity-score model has been correctly specified.

## 6. DISCUSSION

In the current paper we have described methods to assess the adequacy of the specification of the propensity-score model in the context of 1:1 matching on the propensity score. These methods are based on the propensity score being a balancing score: conditional on the propensity score, treated and untreated subjects will have similar distributions of observed baseline covariates. As suggested by others, one can assess the specification of the propensity-score model by examining whether matching on the propensity score balances baseline covariates between treated and untreated subjects [12]. The methods described in the current paper range from those based on comparing means and prevalences of single covariates between treated and untreated subjects to graphical comparisons of the distribution of continuous variables between treated and untreated subjects. The objective of these diagnostics is to determine whether the distribution of observed baseline covariates is similar between treated and untreated subjects matched on the estimated propensity score. Thus, balance diagnostics serve as a test of whether the propensity-score model has been adequately specified.

In RCTs, randomization will balance, in expectation, both measured and unmeasured covariates between the different treatment arms. Senn writes that, in a RCT, ‘over all the randomizations the groups are balanced; and that for a particular randomization they are unbalanced’ [35]. Thus, while randomization will, on average, balance covariates between treated and untreated subjects, it need not do so in any particular randomization. Our discussion of the sampling distribution of the standardized difference in Section 3.3 illustrates that in small RCTs, one would expect modest imbalance in some baseline covariates. In the context of RCTs, several authors have advocated that regression analysis be used to obtain estimates of treatment effect after adjusting for potential imbalance in prognostically important baseline covariates [35–38].

Regression analysis can be used within the propensity-score matched sample to adjust for residual imbalance in observed baseline covariates. However, there are limitations to this approach. An unadjusted estimate is a marginal (or population-average) measure of treatment effect, while the adjusted estimate is a conditional (or adjusted) estimate of treatment effect. When the outcome is continuous and a linear treatment effect is used, then the marginal and conditional treatment effects coincide [39]. However, when the outcome is binary or time-to-event in nature, then marginal and conditional treatment effects do not coincide for non-null treatment effects [39]. Reviews of the use of propensity-score methods in the medical literature have found that they are most frequently used to estimate the effect of treatment on binary or time-to-event outcomes, and are rarely used for continuous outcomes [6]. Similarly, in published RCTs in the medical literature, binary and time-to-event outcomes are more common than are continuous outcomes [40]. In the context of binary outcomes, the use of an adjusted analysis would most frequently be accomplished using a logistic regression, with the odds ratio being used as the measure of treatment effect. The odds ratio has been criticized by several clinical commentators who have suggested that the absolute risk reduction (and the associated number needed to treat) as well as the relative risk are more relevant for clinical decision making than is the odds ratio [41–46]. Both the absolute risk reduction and the relative risk can be computed directly in the propensity-score matched sample. Thus, while the use of regression adjustment can remove the effects of residual imbalance in baseline covariates, its usefulness is tempered by the introduction of the odds ratio as the measure of treatment effect. However, when the outcome is continuous, and the treatment effect is linear, then the use of regression adjustment within the propensity-score matched sample may be particularly useful.

In propensity-score matching there are two competing issues at play. First, even if the true propensity score were known, matching on the propensity score would only in expectation produce balance between treated and untreated subjects in the matched sample. However, a particular matched sample may be subject to imbalance, just as a particular RCT may be subject to imbalance. Our examination of the sampling distribution of the standardized difference in Section 3.3 illustrates that even if the propensity-score model is correctly specified, one can still observe modest standardized differences for baseline covariates. Second, in practice the true propensity score is not known. Thus, residual systematic differences between treated and untreated subjects may be reduced by improving the specification of the propensity-score model. The quandary for the applied researcher is to determine to what degree observed differences between treated and untreated subjects in the matched sample represent a property of that specific sample (which would be observed even if the true propensity score were known) and to what degree observed differences are indicative of the fact that the propensity-score model has been mis-specified. We suggest that modifications of the propensity-score model be attempted with the objective of having the estimated standardized differences for observed baseline covariates lying within the 2.5th and 97.5th percentiles of the empirical sampling distribution of standardized difference in the appropriate sized sample. In small samples, despite heroic attempts to modify the propensity-score model, one may not be able to have all estimated standardized differences below some arbitrary threshold such as 0.25 (25 per cent) or 0.10 (10 per cent). However, the goal in modifying the propensity-score model should be to have the estimated standardized differences lie below the threshold that is consistent with the propensity-score model having been correctly specified. In small samples, it may be necessary to use regression adjustment to remove the effect of residual imbalance of measured confounders.

In addressing the question of observed differences in baseline covariates between groups in the matched sample, both Ho *et al.* and Imai *et al.* suggest that imbalance should be minimized without limit [12, 29]. A difficulty with this approach is highlighted in the previous paragraph: even if the true propensity score were known, it is likely that a certain degree of residual imbalance would be observed. In Section 3 we described methods to determine the empirical sampling distribution of the standardized difference under the assumption that the propensity-score model had been correctly specified. For modest sample sizes, one could expect standardized differences that exceed 0.20 (20 per cent) even when the propensity-score model was correctly specified. The only way to eliminate all imbalance is by matching on all covariates. However, when the number of covariates is large, this is likely to either not be feasible or to result in a dramatically reduced sample size. It is to address this limitation of matching that propensity-score methods were developed.

In our empirical study of different methods for assessing balance, we observed that when we relied on visual comparison of means and proportions, and quantified differences between groups in the matched sample using standardized differences, we came to the conclusion that treated and untreated subjects in the matched sample were similar, with only negligible differences (standardized difference ≤0.030). However, when we compared ratios of the variances of continuous variables between treated and untreated subjects, it was evident that the variance of the distribution of some covariates differed between treated and untreated subjects in the matched sample. Similarly, when we used graphical displays to compare distributions, we observed that the distribution of age differed to a minor degree between the two groups. In particular, differences were most evident in the upper half of the distribution. These differences were masked when only means were compared. These observations suggest that one should consider a range of diagnostics when comparing balance in observed baseline covariates between groups in the matched sample.

We now summarize our recommendations for the use of balance diagnostics in propensity-score matched samples. First, descriptive statistics should always be reported comparing the means of continuous covariates and the frequency distribution of categorical variables between treated and untreated subjects in the matched sample. These are most easily communicated in a table comparing baseline characteristics between treated and untreated subjects in the matched sample. Second, standardized differences should also be reported comparing the means and prevalences of continuous and dichotomous variables between treated and untreated subjects in the matched sample. Third, variances of continuous variables should be compared between treatment groups in the matched sample. Alternatively, one can use standardized differences to compare the means of squared terms of these variables (this is equivalent to comparing second order moments of that variable). For both approaches, one can determine the range of variance ratios or standardized differences that are consistent with the propensity-score model having been adequately specified. The use of five-number summaries may serve as a rough guide for assessing imbalance in baseline covariates; however, its use is limited by difficulty in assessing the amount of variation that one would expect if the propensity-score model were correctly specified. Fourth, the means of two-way interactions between baseline covariates can be compared between treated and untreated subjects. Fifth, the use of quantile–quantile plots of selected, prognostically important covariates can provide further evidence of whether the propensity-score model has been correctly specified. This approach can be seen as a complement to comparisons of variances or second order moments. Sixth, methods based on comparing the distribution of the estimated propensity score between treatment groups are of limited use and provide little information as to whether the propensity score has been correctly specified. The diagnostics described in the current paper have been proposed for assessing whether the propensity-score model has been adequately specified in the context of 1:1 matching on the propensity score. Many of these methods may be modified to the context of many-to-one matching on the propensity score by the inclusion of sample weights, as described elsewhere [47].

In summary, we have described diagnostics for assessing whether the propensity-score model has been adequately specified when using propensity-score matching. Implementing multiple complementary methods for assessing balance allows researchers to better determine whether the propensity-score model has been adequately specified, and thus determine the degree to which matching on the estimated propensity score has reduced or eliminated systematic differences between treated and untreated subjects.
